# Supplementary material for: Factors Associated with Significant Weight Loss in Hospitalised Patients with COVID-19: A Retrospective Cohort Study in a Large Teaching Hospital
Source: Nutrients. 2022 Oct 8;14(19):4195. doi: 10.3390/nu14194195 (PMC9572292; doi:10.3390/nu14194195)
Supplement: Supplementary file 1 [file nutrients-14-04195-s001.zip › nutrients-1938978-supplementary.pdf]

nutrients

MDPI

Supplementary Materials

Factors Associated with Significant Weight Loss in Hospitalised Patients with COVID-19: A Retrospective Cohort Study in a Large Teaching Hospital

Dimitra Zannidi [1], Pinal S. Patel [1], Eleni Leventea [1], Jessica Paciepnik [1], Frances Dobson [1], Caroline Heyes [1], Robert J. B. Goudie [2], Linda M. Oude Griep [3], Jacobus Preller [4] and Lynsey N. Spillman [1,3,*]

SUPPLEMENTARY TABLES

Table S1. Risk-factors for ≥10% weight loss: Univariable binary logistic regression, variables adjusted for age.

Variables	≥10% weight loss during admission	
	**OR, (95% CI)**	**P value**
Age on admission (years)	0.99 (0.97-1.01)	0.461
Sex (male)	1.50 (0.78-2.88)	0.218
Ethnicity (white)	0.91 (0.45-1.83)	0.788
Length of hospital stay (weeks)	**1.29 (1.16-1.42)**	**<0.001**
ICU admission	**3.69 (1.81-7.54)**	**<0.001**
Readmitted	0.86 (0.39-1.89)	0.711
Discharged deceased	0.46 (0.19-1.14)	0.092
CRP on admission(mg/dl)	0.999 (0.995-1.003)	0.546
CRP ≥178 mg/dl during admission	**1.90 (1.01-3.56)**	**0.046**
ISARIC4C Mortality Score (% Mortality)	1.18 (0.30-4.66)	0.814
SpO2/FiO2 ratio on admission	**0.997 (0.995-0.999)**	**0.010**
Urea on admission (mmol/l)	1.02 (0.99-1.05)	0.106
Respiratory rate on admission (breaths/min)	0.96 (0.91-1.01)	0.157
T2DM	1.12 (0.57-2.18)	0.741
Hypertension	0.86 (0.46-1.59)	0.632
Obesity	1.50 (0.57-3.98)	0.415
Chronic neurological conditions	**2.01 (1.09-3.71)**	**0.025**
Weight on admission (kg)	**1.02 (1.00-1.03)**	**0.013**
Total dietetic inputs during admission	**1.06 (1.03-1.10)**	**<0.001**
ONS during admission	1.90 (0.96-3.76)	0.067
Artificial feeding during admission	**2.54 (1.29-5.03)**	**0.007**
SLT assessment during admission	**4.10 (2.21-7.63)**	**<0.001**
Dysphagia on/during admission	**2.49 (1.35-4.61)**	**0.004**
Anorexia on/during admission	1.28 (0.67-2.42)	0.454
BMI≥25 kg/m^2 on admission	1.74 (0.90-3.35)	0.097
Malnutrition screening tool score ≥6 on admission	**2.20 (1.18-4.10)**	**0.013**

Values present OR (95% CI) for each age-adjusted covariate. OR: odds ratio, 95% CI: 95% confidence interval. Bold font: statistically significant results (p<0.05). Abbreviations: GCS: Glasgow Coma Scale; ICU: intensive care unit; BMI: body mass index, CRP: C - reactive protein; T2DM: type 2 diabetes mellitus; ONS: oral nutritional supplement, SLT: speech and language therapist.

Table S2. Risk factors for ≥10% weight loss: Multivariate binary logistic regression with nutrition-related variables included.

Variables	≥10% weight loss during admission	
	**OR, (95% CI)**	**P value**
Age on admission (years)	1.00 (0.98-1.03)	0.796
Sex (male)	1.28 (0.59-2.80)	0.530
Length of hospital stay (weeks)	**1.32 (1.09-1.61)**	**0.005**
ICU admission	1.34 (0.44-4.03)	0.603
CRP ≥178 mg/dl during admission	1.09 (0.50-2.38)	0.826
Weight on admission (kg)	1.06 (0.96-1.17)	0.235
Dysphagia on/during admission	0.91 (0.36-2.29)	0.837
SpO2/FiO2 ratio on admission	0.99 (0.99-1.00)	0.382
Chronic neurological conditions	0.96 (0.44-2.11)	0.918
Malnutrition screening tool score ≥ 6	1.73 (0.74-4.05)	0.207
Total dietetic inputs during admission	0.95 (0.87-1.04)	0.270
ONS during admission	1.50 (0.59-3.81)	0.393
Artificial feeding during admission	0.92 (0.28-2.97)	0.887

Values present OR (95% CI) for all variables included in multivariate logistic regression, OR: odds ratio, 95% CI: 95% confidence interval. Bold font: statistically significant results ($p<0.05$). Total number of patients included in the analysis n= 214, ≥10% weight loss during admission n=39, <10% weight loss during admission n=175. Abbreviations: ICU: intensive care unit, CRP: C reactive protein, ONS: oral nutritional supplement; Comorbidity chronic neurological conditions include ICD-10-CM Codes G00-G99: Diseases of the nervous system; artificial feeding during admission includes any of: NG, NJ, PN, PEG/RIG. OR for Weight on admission is expressed for every 5kg of weight increase, OR for length of hospital stay is expressed per 1 week.

Table S3. Factors associated with more than one dietetic input after discharge: Univariable binary logistic regression models, variables adjusted for age.

Variables	Required more than one dietetic input after discharge (>1 call from CUH dietitians or referral to community dietitians)	
	**OR (95% CI)**	**P value**
CRP ≥178 mg/dl during admission	**2.14 (1.22-3.78)**	**0.008**
SpO2/FiO2 ratio on admission	1.00 (0.99-1.00)	0.508
≥10% weight loss during admission	**2.65 (1.33-5.28)**	**0.006**
Length of hospital stay (weeks)	1.06 (0.99-1.14)	0.075
ICU admission	**2.03 (1.05-3.92)**	**0.036**
Artificial Feeding during admission	**2.24 (1.19-4.23)**	**0.012**
SLT assessment during admission	1.68 (0.92-3.08)	0.090
T2DM	1.05 (0.57-1.93)	0.878
Obesity	0.27 (0.06-1.16)	0.079
Hypertension	1.06 (0.60-1.88)	0.829
Chronic Neurological Conditions	1.27 (0.70-2.29)	0.430

OR: odds ratio, 95% CI: 95% confidence interval. Bold font: statistically significant results ($p<0.05$). Abbreviations: ICU: intensive care unit, CRP: C-reactive protein, SLT: speech and language therapist, T2DM: type 2 diabetes mellitus.

Table S4. Factors associated with ONS GP prescription request: Univariable binary logistic regression models, variables adjusted for age.

**Variables**	**Required ONS GP prescription request**	
	**OR (95% CI)**	**P value**
CRP ≥178 mg/dl during admission	1.49 (0.72-3.08)	0.278
SpO2/FiO2 ratio on admission	1.00 (0.99-1.01)	0.259
≥10% weight loss during admission	1.13 (0.43-2.99)	0.802
Length of hospital stay (weeks)	1.01 (0.91-1.11)	0.893
ICU admission	0.89 (0.36-2.19)	0.802
Artificial feeding during admission	1.04 (0.44-2.98)	0.925
SLT assessment during admission	0.95 (0.41-2.20)	0.904
T2DM	0.82 (0.35-1.89)	0.636
Obesity	0.00 (0.00-0.00)	0.998
Hypertension	1.50 (0.71- 3.19)	0.290
Chronic Neurological Conditions	0.76 (0.33-1.74)	0.512

OR: Odds ratio, 95% CI: 95% confidence interval. Bold font: statistically significant results ($p<0.05$). Abbreviations: ICU: intensive care unit, CRP: C-reactive protein, SLT: speech and language therapist, T2DM: type 2 diabetes mellitus.
